# Author Correction: Microbial spatial footprint as a driver of soil carbon stabilization

**DOI:** 10.1038/s41467-019-12000-3

**Published:** 2019-09-05

**Authors:** A. N. Kravchenko, A. K. Guber, B. S. Razavi, J. Koestel, M. Y. Quigley, G. P. Robertson, Y. Kuzyakov

**Affiliations:** 1 Department of Plant, Soil and Microbial Sciences, Michigan State University, East Lansing, MI 48824 USA; 2 DOE Great Lakes Bioenergy Research Center, Michigan State University, East Lansing, MI USA; 3 Department of Agricultural Soil Science, University of Göttingen, Göttingen, Germany; 4 Department of Soil and Plant Microbiome, Institute of Phytopathology, Christian-Albrecht-University of Kiel, Kiel, Germany; 5 Swedish University of Agricultural Sciences, Uppsala, Sweden; 6 W. K. Kellogg Biological Station, Michigan State University, Hickory Corners, MI 49060 USA; 7 Institute of Physicochemical and Biological Problems in Soil Science, 142290 Pushchino, Russia; 8 RUDN University, Moscow, Russia

**Keywords:** Agriculture, Biogeochemistry, Environmental sciences

Correction to: *Nature Communications* 10.1038/s41467-019-11057-4, published online 16 July 2019.

The original version of this Article omitted the following from the end of the Acknowledgements: ‘We gratefully acknowledge the German Research Foundation (DFG) for supporting the project: RA3062/3-1.’ This has now been corrected in both the PDF and HTML versions of the Article.

