# Supplementary material for: Microbial spatial footprint as a driver of soil carbon stabilization
Source: Nat Commun. 2019 Jul 16;10:3121. doi: 10.1038/s41467-019-11057-4 (PMC6635512; doi:10.1038/s41467-019-11057-4)
Supplement: Supplementary file 1 — Supplementary Information [file 41467_2019_11057_MOESM1_ESM.pdf]

Microbial spatial footprint as a driver of soil carbon stabilization

Kravchenko et al.

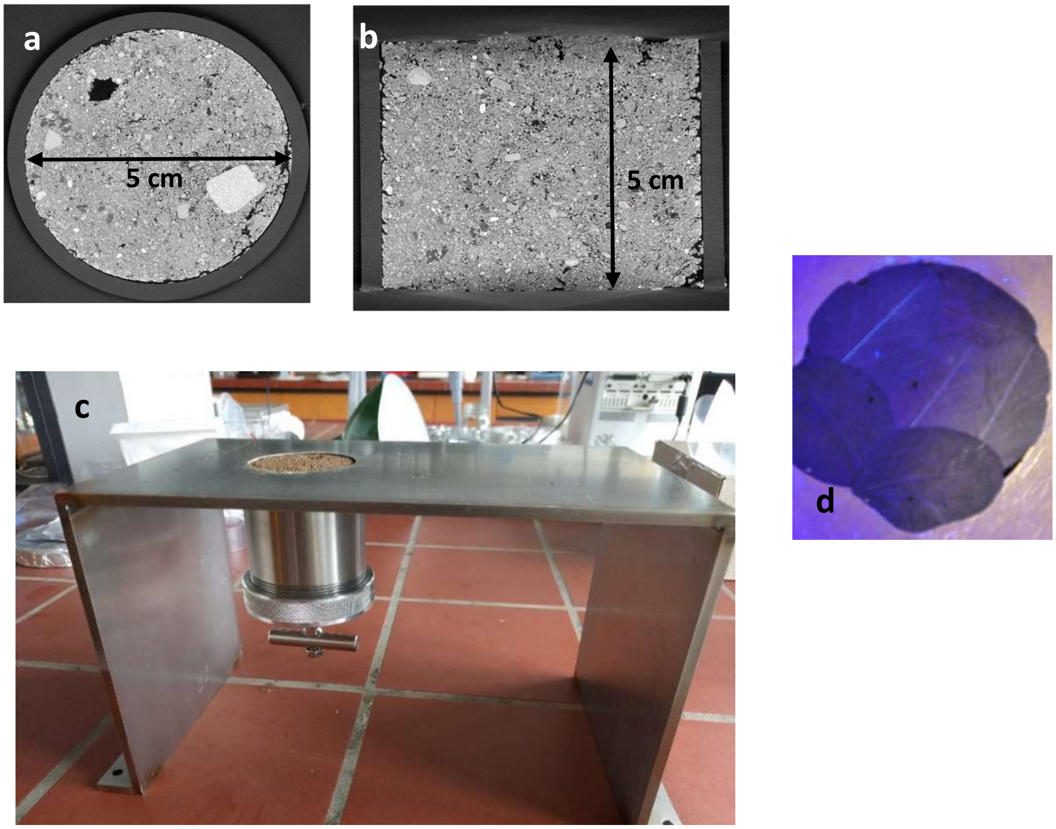

Supplementary Fig. 1. Illustration of the experimental set up. Example of **a** top and **b** front view of an X-ray μCT scanned soil core. **c** Experimental set up used to cut soil layers from X-ray μCT scanned soil cores. **d** Layer of clover leaves incubated on the surface of the soil sample.

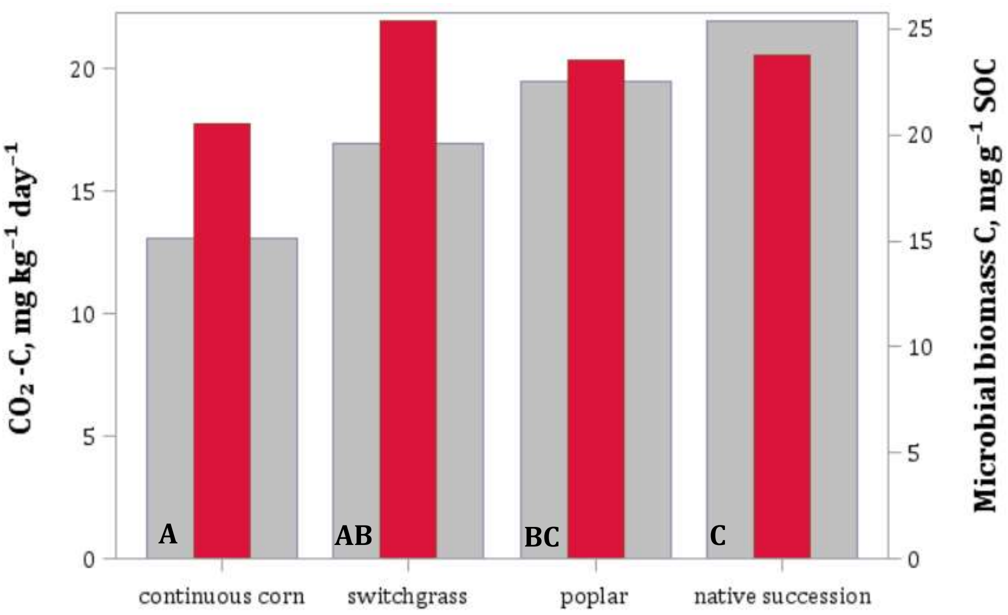

Supplementary Fig. 2. Short-term respiration and microbial biomass C. Cumulative amount of CO_2-C respired during short-term (7 day) incubation (gray) and microbial biomass C expressed per unit of soil organic C (red). Letters mark statistically significant differences among the cropping systems in terms of short-term respiration ($p<0.05$). Standard errors are equal to 2.0 mg kg^{-1} day^{-1} and 2.1 mg g^{-1} for CO_2-C and microbial biomass C, respectively. Source data are provided as a Source Data file.

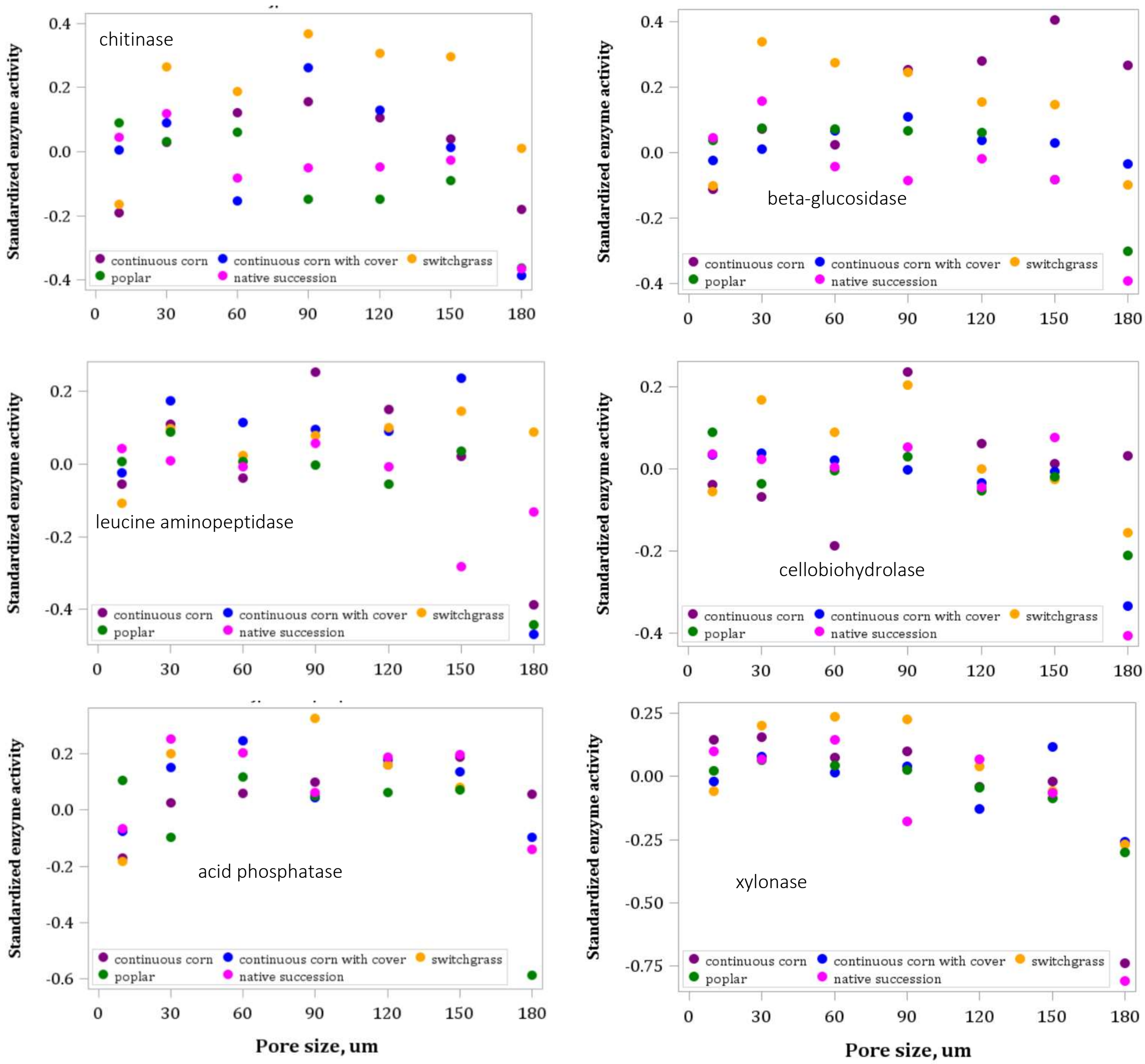

Supplementary Fig. 3. Standardized activities of the studied enzymes. Standardized enzyme activities in soil localities with prevalence of pores of different sizes from soil slices not subjected to incubations with fresh C inputs. Shown are means for all studied enzymes. Summary data are provided as a Source Data file.

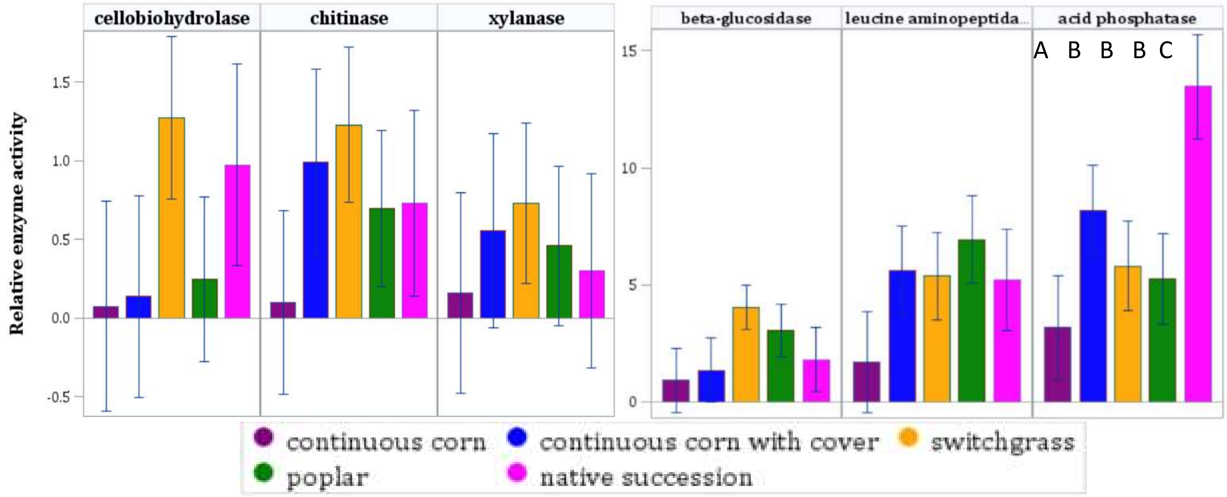

Supplementary Figure 4. Relative enzyme activities in the studied cropping systems. Letters mark statistically significant differences among the systems within specific enzymes ($p<0.05$). Errors bars represent s.e.m. Note that y-axis scales are different for cellobiohydrolase, chitinase, and xylanase, from that of the other three enzymes due to substantial differences in their activities and the need to visualize the differences among the systems on the graph. Summary data are provided as a Source Data file.

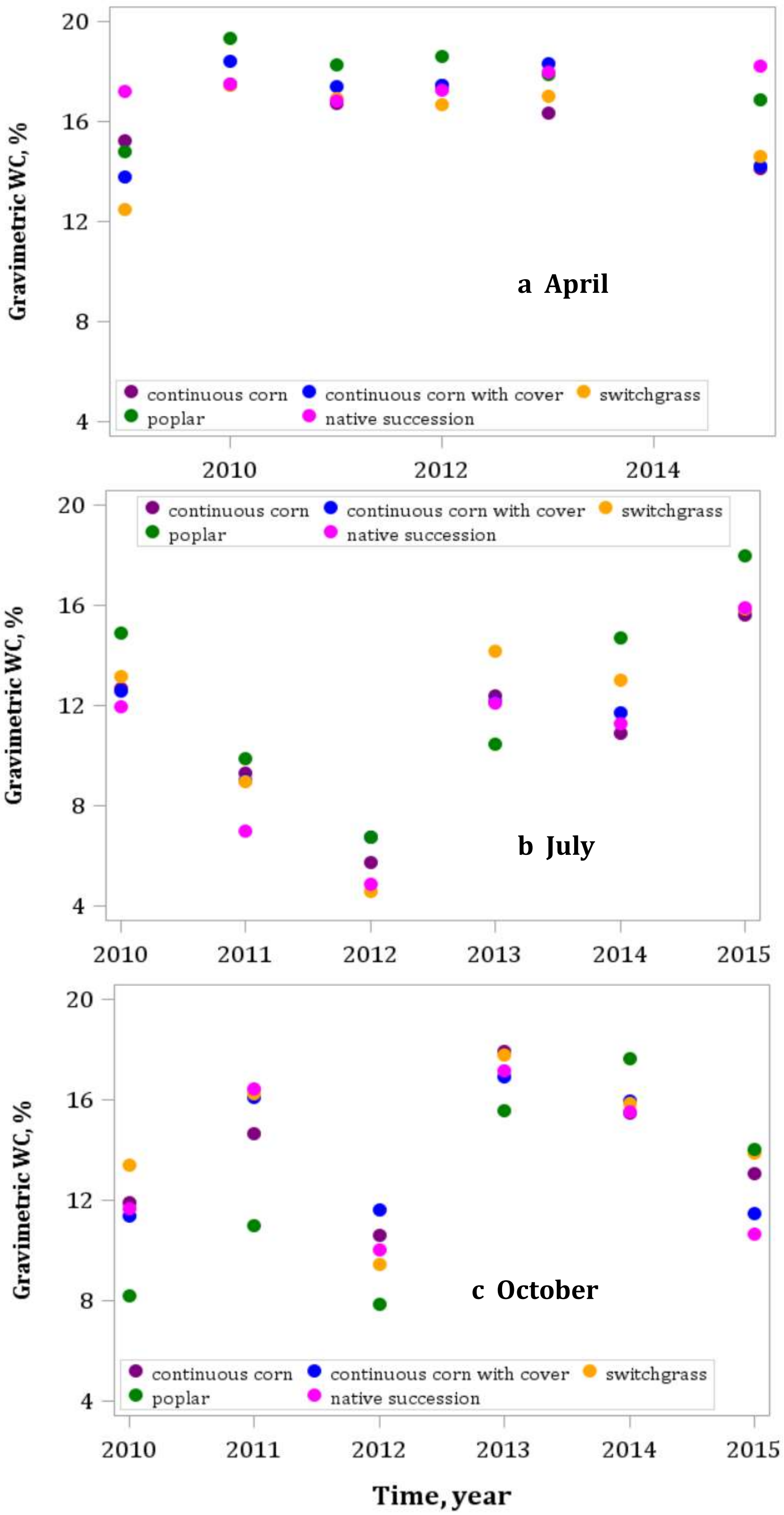

Supplementary Fig. 5. Soil moisture monitoring results. Monthly averages of soil gravimetric water content (WC) at 0-25 cm depth from 2009 to 2015 for (**a**) April, (**b**) July, and (**c**) October. Source data are provided as a Source Data file.

Supplementary Fig. 6. A set up for combining zymography with X-ray µCT information. The experimental set up used for zymography analyses of intact soil cores (**a**) and a schematic representation of the soil sample processing procedures for joint pore-size distribution and relative enzyme activity analyses (**b**).

a)

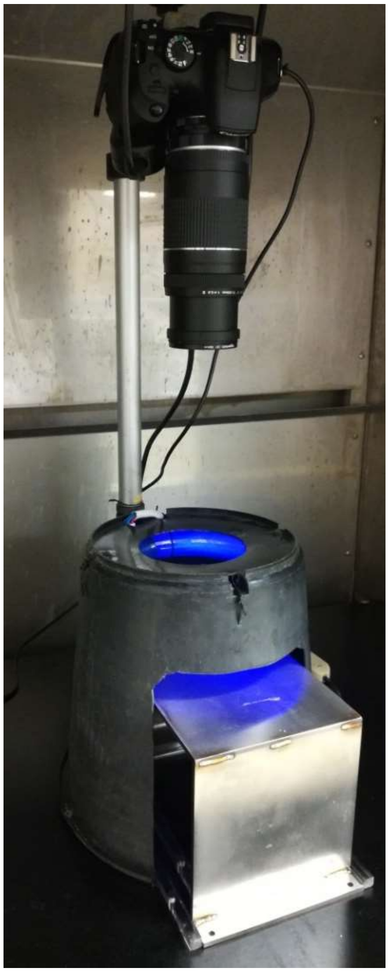

b)

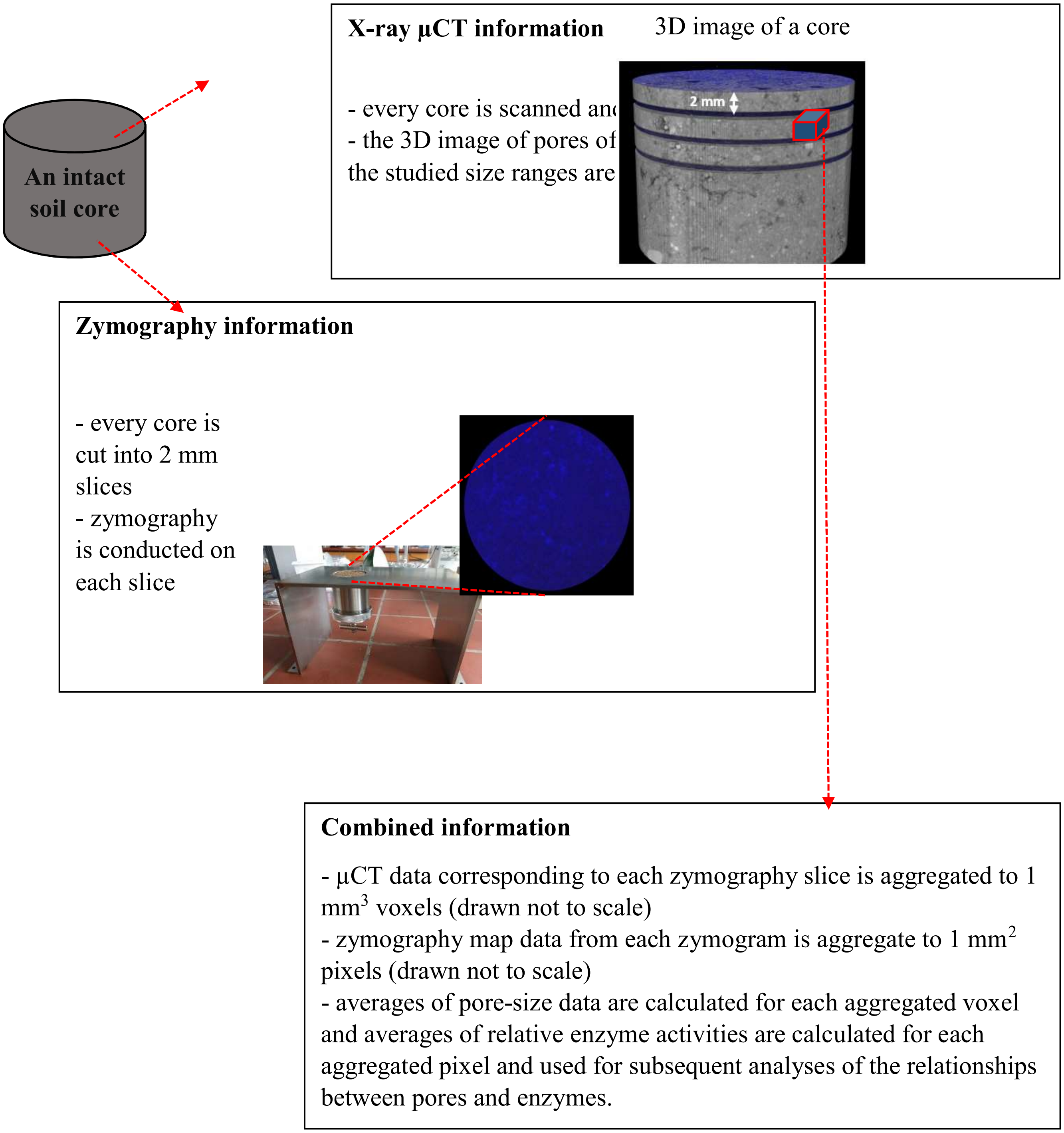
An intact soil core
X-ray µCT information
3D image of a core
- every core is scanned an
- the 3D image of pores of
the studied size ranges are
2 mm
Zymography information
- every core is cut into 2 mm slices
- zymography is conducted on each slice
Combined information
- µCT data corresponding to each zymography slice is aggregated to 1 mm3 voxels (drawn not to scale)
- zymography map data from each zymogram is aggregate to 1 mm2 pixels (drawn not to scale)
- averages of pore-size data are calculated for each aggregated voxel and averages of relative enzyme activities are calculated for each aggregated pixel and used for subsequent analyses of the relationships between pores and enzymes.

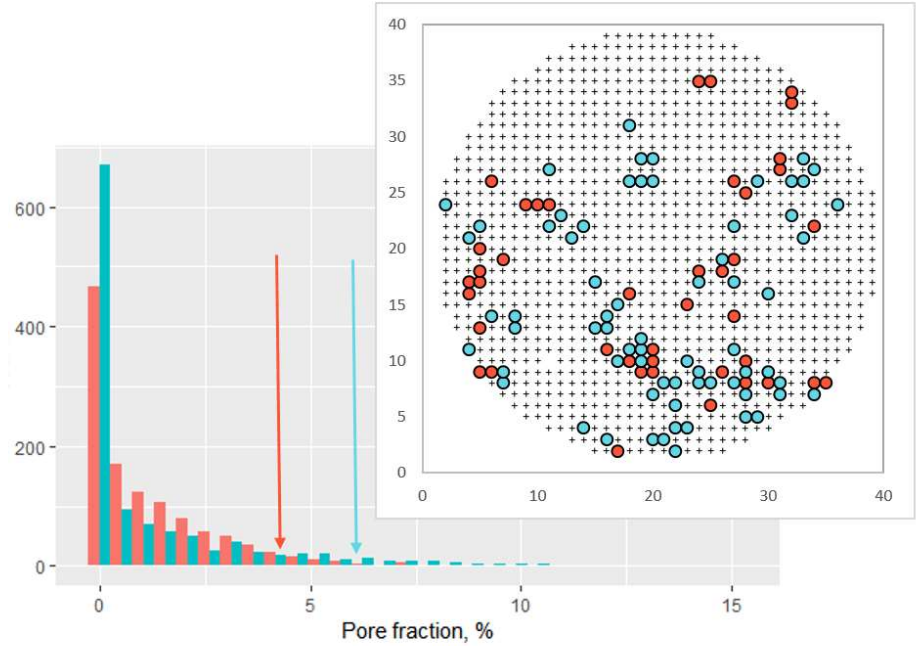

Supplementary Fig. 7. **Illustration of pore data preparation**. Examples of histograms for abundances of pores in 60 µm (red) and 90 µm (blue) size range from one of the studied cores. Arrows mark locations of the respective 95th percentiles for these two pores sizes in the core. The insert shows the voxels where the abundances of these pores exceeded the respective 95th percentiles in one of the 2D zymography layers, and which were used in the subsequent analyses of the associations between pores and enzyme activities.

Supplementary Table 1. Standardized enzyme activities in soil localities with prevalence of pores of different sizes from soil slices not subjected to incubations with fresh C inputs. Shown are means, standard errors and letters marking significant differences among cropping systems in localities with prevalence of pores of different size classes ($p<0.05$) across all studied enzymes.

	**Pore size class**						
**Cropping system**	**<30 µm**	**30 µm**	**60 µm**	**90 µm**	**120 µm**	**150 µm**	**>180 µm**
**continuous corn**	-0.07/0.03ab	0.05/0.04a	0.01/0.04a	0.18/0.04cd	0.12/0.04bc	0.11/0.04b	-0.16/0.03c
**continuous corn with cover**	-0.02/0.02b	0.09/0.04a	0.05/0.04a	0.09/0.04bc	0.05/0.04abc	0.09/0.03b	-0.26/0.03b
**switchgrass**	-0.11/0.02a	0.21/0.03b	0.17/0.03b	0.24/0.04d	0.13/0.03c	0.10/0.03b	-0.09/0.03c
**poplar**	0.06/0.02c	0.02/0.03a	0.05/0.03a	0.00/0.03ab	-0.03/0.03a	-0.03/0.03a	-0.37/0.03a
**native succession**	0.03/0.02c	0.11/0.04a	0.04/0.04a	-0.02/0.04a	0.02/0.04ab	-0.03/0.04a	-0.37/0.03a
